# Improving communication between doctor and patient: eHealth in the Netherlands, an established cloud solution

**Published:** 2012-06-15

**Authors:** Anton Kool

**Affiliations:** Curavista BV, The Netherlands

**Keywords:** eHealth, cloud solution

## Abstract

In the Netherlands, like in many West European countries, demand for healthcare is already sharply increasing, with further acceleration expected soon. All parties involved are convinced that the resulting demand for funding of healthcare will not be met by economic growth. The resulting paradigma shift (live longer healthy, self-care and patient centred care) is a challenge not only for scientists, but for politicians and healthcare-providers as well.

One of the solutions in the paradigma shift is eHealth. eHealth can refer to automated data-exchange between a device and a central database, but also to healthcare practices that use webbased communication. Strengthening patient participation, motivation and self-management is the hope for better therapy outcome. Early deviations need to be recognized, adverse reactions to be understood and appropriate action to be taken. In itself not new, diaries have been around for decades, but appropriate assessment of its content is too time-consuming. Therefore, the challenge is to involve both the patient and the attending professional (-s) and give eHealth solutions a place in the context of regular care. We combined the internet cloud with advanced security-technology to provide an answer to that: Curavista health, a database driven internetplatform for patient@home and doctor@work.

Patient@home replies to webquestionnaires and fill online diaries. The responses are summarized in tables, graphs or automated follow-ups and the patient has immediate insight in the progression achieved. Not only does database technology allow for immediate processing of the responses into summaries; it is also possible to highlight differences, produce alerts or (refer to) educational information.

Doctor@work, using an own account, has access to the responses as well as to the summaries, resulting in early insight. Because the patient@home does not necessarily record only biometrics, but also has the opportunity to add other types of replies, which gives a broader context to the actual situation the patient is in.

Regular care is not only faced with an ageing population but, as a result of new interventions, also with an increased incidence of people with multiple diseases. Curavista health has therefore been designed with the process of chronic care as its cornerstone: the basic architecture for each indication is identical, the content is different. Multiple indications can be attained to one account (doctor or patient) and multiple relations (doctor to patients, patient to professionals) maintained.

Started in 2002, today more than 20 indications are covered using this platform. It is used in both primary care (GPs) as well in out-clinic hospital care: 1000+doctors monitor(-ed) 50,000+patients. The first reports are that face-to-face consultations not only run more effective and more efficient when using the summaries, but also more satisfactorily: the available time can be devoted to the patient need, rather than to the inventory of the patient needs. Is it only a matter of time until timing of face-to-face consultations can be based upon cloud-based eHealth solutions?

